# Development of a Personal Ultrasound Exposimeter for Occupational Health Monitoring

**DOI:** 10.3390/ijerph182413289

**Published:** 2021-12-16

**Authors:** Michal Cieslak, Christoph Kling, Andrea Wolff

**Affiliations:** 1 Physikalisch-Technische Bundesanstalt (PTB), 38116 Braunschweig, Germany; Christoph.Kling@ptb.de; 2 Institut für Arbeitsschutz der Deutschen Gesetzlichen Unfallversicherung (IFA der DGUV), 53757 Sankt Augustin, Germany; Andrea.Wolff@dguv.de

**Keywords:** occupational health, personal sound exposimeter, sound level measurement, ultrasound

## Abstract

Prolonged exposure to airborne ultrasound in a workplace can have a detrimental influence on a worker’s well-being. Given the ever-increasing use of ultrasonic industrial equipment, it is of vital importance—and may also be regulated by law—to monitor ultrasound exposure during a normal workday as part of workplace risk assessment. However, the devices currently utilized exhibit limitations with regard to both their operational frequency and their portability (wearability). In this paper, the first prototype of a high-frequency and ultrasound personal exposimeter is presented in the light of the latest national and international standards governing high-frequency and ultrasonic noise measurement in the field of occupational health monitoring. The prototype was tested in the laboratory environment in order to assess its sound level detection capabilities in both the audible and ultrasonic frequency ranges. Several common industrial scenarios—including an ultrasonic welding machine, an ultrasonic cleaning bath, and a compressed air gun—were simulated in a laboratory environment. For each simulated set-up, a corresponding high-frequency or ultrasonic signal was fed through a specially prepared generation chain. Each experimental scenario was initially surveyed with an ultrasound level meter previously tested up to 100 kHz. This was followed by a measurement with the prototype. For this study, the simulated sound signals varied between 10 kHz and 40 kHz on the frequency scale and between 60 dB and 90 dB in amplitude. The portability of the prototype, which may be required to be worn throughout an entire workday (e.g., 8 h), was also considered. All the experiments were performed on a customized ultrasound measurement set-up within a free-field environment located at the Physikalisch-Technische Bundesanstalt (PTB) in Braunschweig, Germany. Results obtained suggest a good agreement between the measurements performed with both devices in the louder areas of the sound fields produced. Because the overall measurement uncertainty is highly dependent on the specificity of the individual measurement set-up and measurement procedure, an uncertainty budget estimated for the prototype considers electro-acoustical contributions only.

## 1. Introduction

With regard to audible sound, strategies for measuring workplace exposure are primarily provided by the ISO 9612:2009 [1] standard. This standard states that measurements can be performed with either a hand-held sound level meter or a personal sound exposimeter, specifications for which are defined in IEC 61672-1:2013 [2] and IEC 61252:1993 [3], respectively. The choice of instrument depends on the circumstances of a particular measurement scenario and should be made by the technician performing the measurement.

There are, however, an increasing number of industrial appliances that produce noise in the high-frequency and ultrasonic range. These include machines such as ultrasonic cleaning baths, welding machines, drills, soldering guns, cutting machines, etc., [4,5]. Although many countries, such as Poland, have included ultrasonic noise exposure in a workplace in their risk assessment, there are no defined international regulations specifying suitable measurement instruments or methods [6]. Moreover, the threshold between high-frequency and ultrasonic noise differs among countries [7].

In order to perform such measurements, sound level meters and microphones meeting the requirements of Class 1 specifications (IEC 61672-1:2013 [2] and IEC 61094-4:1995 [8]) are needed. It is also necessary to select both the microphone and the measuring device itself to suit the frequency range of interest for the particular measurement. However, according to the German national guideline for the measurement of ultrasound at the workplace (VDI 3766:2012 [4]), the frequency range of the measuring instrument must reach at least 40 kHz (one-third octave band center frequency). Furthermore, occupational health monitoring regulations require that such devices—as well as personal sound exposimeters—be subjected to periodic conformance tests in accredited approval centers.

Extensive analysis of the requirements for measuring ultrasound in a work environment, together with suggested adaptations of currently available methods for audible sound measurements, have been presented by Radosz and Pleban [9]. Their work identified a number of other issues, such as the lack of clear information in literature on the ultrasonic noise uncertainty budget or on the factors which may affect sound pressure level (SPL) measurement. Moreover, a recently published Polish national standard provides a detailed description of the equipment and measurement process required for the assessment of airborne ultrasound exposure at the workplace in Poland [10]. In line with the cited international standards and the German national guideline, the Polish national standard defines the requirements for measurements between 10 kHz and 40 kHz (one-third octave band center frequency).

### 1.1. Occupational Ultrasound Measurement

There are no internationally defined thresholds between audible, high-frequency and ultrasonic noise. However, there are examples in the literature of these boundaries being defined on the national level. For instance, the ultrasonic spectrum defined in Poland includes the high audible frequencies and spans the range from 10 to 40 kHz. In France, on the other hand, there is a clear distinction between high-frequency audible sound (8–20 kHz) and low-frequency ultrasound (20–50 kHz) [11]. In the United Kingdom, an often-found distinction is placed at 17.8 kHz, with frequencies between 11.2 and 17.8 kHz referred to as very high-frequency sound (VHFS) and the term ultrasound used for frequencies above 17.8 kHz [12]. In Germany, audible sound is separated from ultrasound at 16 kHz. For the purposes of this work, sounds of frequencies above 16 kHz will therefore be classified as ultrasounds.

Regardless of its classification, ultrasound in air has been associated with adverse and recurring health effects. Recent studies have attempted to deal with the biological, mechanical and general effects of exposure to low-frequency ultrasound (20–100 kHz) on human beings and have examined the difficulties that still exist in determining ultrasound exposure [13,14,15]. Notwithstanding these challenges, the effects of exposure have been divided into four groups: subjective symptoms of exposure to ultrasonic noise, impact on hearing, thermal effects, and functional changes [11].

Subjective symptoms such as headaches, migraines, nausea, and fatigue have been consistently reported by industrial workers operating machinery described as ultrasonic [16,17]. Due to their subjective nature, it is difficult to unequivocally assess such symptoms. Furthermore, ultrasound noise in a workplace is often accompanied by high-level sound in the high-frequency audible range, which can cause similar symptoms. However, an increasing number of studies dealing exclusively with ultrasonic noise suggest a direct link between those symptoms and ultrasound exposure [11].

Prolonged exposure to ultrasound noise may also be linked to hearing threshold shift [17,18]. Although further investigations are required, current literature suggests that regular ultrasound exposure at the workplace is the second most important factor (after age) contributing to the occurrence of hearing level shift [18]. Other studies indicate that ultrasonic noise exposure may lead to increased body temperature as well as to functional changes (irritation, memory problems, concentration and learning difficulties) [11]. Furthermore, recent findings suggest that ultrasound fields can be highly inhomogeneous, meaning that ultrasonic noise exposure may vary depending on individual physiology [19,20].

It is therefore of vital importance not only to identify the sources of ultrasound in a workplace but also to characterize the ultrasonic fields produced by these sources. To date, however, the approach used to achieve this aim has been to attempt to adapt the methods used for audible frequency spectrum characterization. This approach is questionable due to the lack of comprehensive normative regulations for both ultrasound measuring methods and the equipment capable of measuring ultrasonic noise. This problem was cited as far back as the early 1980s by the World Health Organization (WHO), which identified the lack of suitable instrumentation as the reason behind the insufficient characterization of complex exposure parameters within ultrasonic fields [21].

A recently presented measurement procedure allows not only the determination of occupational exposure to ultrasonic noise, but also describes technical requirements placed on the measuring equipment [20,22]. For instance, the VDI 3766:2012 [4] guideline claims that free-field microphones (1/4" or 1/8") are sufficient since they allow the influence of the directivity of the microphone to be kept as low as possible. However, recent findings have revealed large variations in the sound levels occurring between two close points in space—up to 20 dB at points 2.6 cm apart [22]. It is therefore necessary to take account of a worker’s movements in an ultrasonic environment, as individual exposure may vary dramatically due to differences in body height, posture, movement patterns, etc.

Following from that is the need to quantify personal exposure to ultrasound fields. A recently developed ultrasound level meter (the USPM—from German Ultraschall-Pegelmesssystem) was able to successfully measure ultrasonic noise in the work environment up to 100 kHz [23]. The device was built using commercially available components. Despite its reliable measurement performance, a device of this scale is primarily intended for stationary measurements. It would therefore be difficult to take account of a worker’s movements during the measurement.

### 1.2. Personal Ultrasound Exposimeter

Measurement of audible sound exposure in a workplace can be performed using a hand-held sound level meter placed in a defined location at a workstation. Personal exposure can then be established based on the time a worker spends at that location. Alternatively, a personal sound exposimeter can be employed. Such a device can normally be worn throughout the day to account for the worker’s movements during the workday. However, the normative demands for such devices currently only apply up to a limit of 8 kHz (with recommendations given up to 12.5 kHz) [3].

In this work, a prototype of the High-Frequency and Ultrasound Personal Exposimeter (HiFUSPEx) is described. The prototype was built using a combination of custom-designed elements and commercially available parts. Due to specific operational requirements in the ultrasonic frequency range, the acoustical properties of the components were of paramount importance. Power consumption, weight and wearability were also considered. The next two sections describe the development process and provide a detailed description of the complete device. This is followed by a presentation of the experimental setup and methodology used in this work to characterize the HiFUSPEx. The results obtained are then presented and analyzed in the subsequent discussion section, which is followed by a section on uncertainty consideration. In the final section, main conclusions are drawn and possible directions of future development identified.

## 2. Design and Development of The Prototype

### 2.1. Requirements

Given the technical and normative limitations related to ultrasound measurement techniques as described in preceding sections, it was necessary to define a set of requirements for a portable sound measuring system intended for operation in the high-frequency and ultrasonic range. Schöneweiß et al. [22] recently presented a list of five properties, together with comprehensive descriptions of each element, required for the measurement of airborne ultrasound: frequency range, linearity and dynamic range, spatial resolution, signal-to-noise ratio and traceability. It should be noted that the work of Schöneweiß et al. [22] documents an investigation into the characterization of airborne ultrasonic fields.

One of the findings of the study by Schöneweiß et al. [22] is that ultrasound fields are highly inhomogeneous. It is for this reason necessary to be able to investigate workers’ ultrasound exposure individually, something that can only be achieved with great difficulty when standard sound level meters intended for stationary measurements are used. This is why portability (wearability) and connectivity needed to be added to the list of requirements for the development of a personal sound measuring device.

#### 2.1.1. Frequency Range

In order to comply with the aforementioned international standards and the German national guideline, the frequency range of a personal ultrasound exposimeter should at least cover the 40 kHz one-third octave band (center frequency). An extensive investigation of industrial ultrasonic sources in Germany further showed that the operating frequency of such devices typically falls within the range between 16 kHz and 40 kHz [17]. However, it is generally assumed that exposure to low-frequency ultrasound (≤100 kHz) can be associated with negative health effects [15]. Therefore, a frequency range of 10–100 kHz is preferred, as it also covers the first harmonic of the highest and the first subharmonic of the lowest typical operational frequencies of ultrasonic equipment in Germany.

#### 2.1.2. Linearity and Dynamic Range

The cited study by Ullisch-Nelken et al. [17], which was based on 131 measurements performed at sites in Germany associated with ultrasound, suggests that the typically encountered Z-weighted SPL peak values are spread across the range of approximately 70 dB to 150 dB (re 20 μPa). These were measured for different ultrasonic equipment with various operational frequencies. Due to the strong heterogeneity of ultrasonic fields, it is of vital importance that a personal sound exposimeter intended for ultrasound measurements have a high dynamic range. Such a device is capable of differentiating between the sound pressure maxima and minima, which can occur within a very short distance from one another.

#### 2.1.3. Spatial Resolution

The spatial resolution requirement was considered by Schöneweiß et al. [22] based on the scans of various areas around the sound source which were utilized to fully characterize the ultrasound field. For personal sound exposimeters, however, the sound field is not scanned. The sensitive sensor is placed in one location, such as on a worker’s shoulder, and its displacement is governed by the worker’s movements at the workplace. This means that only the size of the microphone’s diaphragm needs to be considered for the personal sound level meter.

With the smaller size of the microphone, the SPL is averaged over a proportionally smaller area. As a result, the spatial resolution of the measurement is increased, and the microphone’s contribution to the overall uncertainty evaluation is reduced. This is especially important in ultrasound fields produced by industrial equipment, e.g., welding machines that emit tonal components of the operating frequency. A displacement of a few centimeters within those fields may lead to SPL differences in the order of tens of dBs, as indicated by Schöneweiß et al. [22], where a median distance of 2.6 cm between the local sound pressure extrema was quoted.

#### 2.1.4. Signal-to-Noise Ratio

In order to ensure a suitable signal-to-noise ratio of approximately 20 dB, the inherent noise of each component of the electro-acoustic measuring chain should be kept as low as possible. For this reason, microphones with higher sensitivity are preferred as they are less likely to be influenced by ambient noise. Inherent level consideration is particularly important for personal sound exposimeters, where an input analog signal may be affected by the internal electronic circuitry of the device. As these must be placed in a relatively small enclosure, the low SPL limits for personal sound exposimeters are often higher than those for corresponding hand-held sound level meters.

#### 2.1.5. Traceability

When performing SPL measurements for the purposes of occupational health monitoring, it must be ensured that the results are as accurate and precise as possible. This can be achieved through the calibration of the measuring system, and is particularly important within the ultrasonic frequency range, where directivity patterns and atmospheric conditions may greatly influence the measurement results. These factors therefore need either to be compensated for or included in the uncertainty consideration.

#### 2.1.6. Portability

As previously mentioned, industrial ultrasonic fields can be strongly heterogeneous. Therefore, individual ultrasound exposure tracking for workers operating ultrasound emitting equipment is preferred. The device to be deployed must therefore be small, lightweight, and designed in such a way that it does not restrict the ability of workers to perform their tasks. It should ideally be waist-mounted, with the microphone attached to the worker’s shoulder in order to avoid reflections from the device’s enclosure.

#### 2.1.7. Connectivity

Given that personal sound exposimeters are, according to ISO 9612:2009 [1], primarily designed for full-day measurements, the device remains attached to the worker’s shoulder after the technician overseeing the measurement has left the measurement area. It would therefore be preferable to have the device’s status monitored remotely. Although the worker whose sound exposure is being monitored is supposed to remain in the technician’s view, such remote monitoring would allow invalid measurements caused, for instance, by unintended switching off, to be detected and acted upon immediately. Immediate feedback would also enable the technician to recognize issues with the measurements in real time and to carry out the necessary corrections.

### 2.2. Schematic (Block) Design of the Prototype

**Figure 1:**
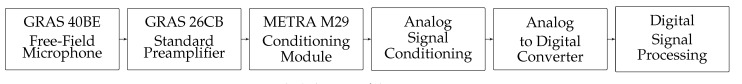
Block diagram of the HiFUsPEx prototype.

A sound exposimeter typically comprises a set of standard building blocks similar to those of a sound level meter. This is also the case with the prototype of the HiFUSPEx, the block diagram of which is presented in Figure 1. An acoustic signal is transduced into an electrical analog signal using a free-field microphone with the appropriate frequency response. The analog signal goes through a pre-amplifier stage and is then further conditioned and adjusted to the inputs of the analog to digital converter (ADC). The ADC is responsible for the conversion of the continuous-time signal into a number of discrete values defined by the ADC’s sampling rate. The discrete-time signal is then processed in the digital domain. At this stage the signal is filtered for the required frequency range and the time and frequency weightings are applied for the specific value to be measured. These are subsequently shown on a digital display in real time.

### 2.3. Microphone & Preamplifier

For the purposes of acousto-electric conversion, the GRAS 46 BE 1/4" Integrated Electronics Piezo-Electric (IEPE) Free-field Standard Microphone Set was employed. It comprises the GRAS 40BE 1/4" Prepolarized Free-Field Microphone and GRAS 26CB 1/4" Constant Current Power (CCP) Standard Preamplifier. This microphone set is designed according to IEC 61094-4:1995 [8] as a working standard 3 (WS3) microphone and is characterized by a flat frequency response between 4 Hz and 100 kHz (±3 dB) and by lower and upper dynamic range limits of 35 dB and 160 dB, respectively. The manufacturer’s data sheet specifies a sensitivity of 3.6 mV/Pa and an output impedance of <50 Ω. Moreover, it requires a constant current input module capable of delivering 4 mA and 24 V of unloaded voltage supply in order to perform according to the specification [24].

Its small dimensions (53 mm in length), and low weight (8 g) make it suitable for portable applications. The GRAS 46BE set comes terminated with a microdot female connector as standard. To connect the microphone set with the enclosure of the prototype, a customized 2-pin microdot-LEMO cable was fabricated. This modification was dictated by the experience gained during previous work with the standard BNC connectors, which proved to be prone to developing discontinuity issues when used in the field. The standard length of the cable (3 m) was chosen to allow adjustment to various measurement scenarios during laboratory testing. It can, however, be optimized for the field-testing stage, where the enclosure will be attached around the worker’s waist and the microphone placed near the worker’s ear.

The microphone set is powered by the M29 Conditioning Module from METRA [25]. The M29 METRA Module can be powered from a 5–28 V and <100 mA supply to provide a constant current output of up to 4.5 mA and a 24 V voltage source. As such, the conditioning module can be supplied from a standard regulated 5 V power supply. To allow easier integration and to make best use of sparse space available in a compact, portable device, only the printed circuit board was extracted from the METRA M29 Module and integrated in the prototype’s enclosure.

### 2.4. Analog to Digital Converter

The ADC, whose characteristics primarily define the overall features of the sound level meter, is the core element of the electrical processing part. The ADC’s operational frequency range and its dynamic range are of particular interest in this type of application. The ADC characteristics responsible for the definition of these features are the sampling rate and the resolution, respectively. For this reason, the frequency response of two commercially available ADCs of equal resolution (24-bit) and different sampling rates were compared prior to the development of the prototype.

The majority of cases involving the monitoring of ultrasonic noise at the workplace reported increased sound levels detected at frequencies of up to 40 kHz (one-third octave band center frequency) [9]. However, current literature suggests that ultrasonic frequencies up to 100 kHz may have detrimental health effects on the human body [7,11]. Therefore, the two ADCs manufactured by Texas Instruments (TI)—ADS127L01 and ADS1672—were tested at the sampling rate of 256 kSPS and 312 kSPS, respectively [26,27].

**Figure 2:**
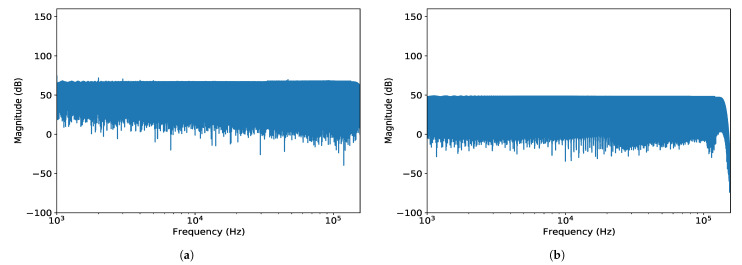
A comparison of the ADC’s response to the sweep signal between 1 Hz and 160 kHz plotted for both converters: (**a**) the response of ADS1672 operating at 312 kSPS and (**b**) the response of ADS127L01 operating at 256 kSPS.

In each case, the responses to an electrical sinusoidal sweep signal between 1 Hz and 160 kHz were recorded and stored as a raw data *.wav file. Readout electronics were implemented using Teensy 3.6 USB Development Board with a Freescale MK66FX1M0VMD18 32-bit 180-MHz ARM Cortex-M4 microcontroller. The averaged response of both ADCs over the recorded period was computed; the resulting frequency responses for the ADS1672 and ADS127L01 are shown in Figure 2a,b, respectively.

ADS1672 maintains a stable magnitude value until its Nyquist frequency at 156 kHz. Similarly, ADS127L01 performs in line with its specification as the roll-off does not begin to appear until 128 kHz. It should also be noted that the expected behavior was also observed when noise floor is considered. With both devices set up for high-resolution performance, there was higher noise floor content in the data collected with ADS1672. Moreover, ADS1672 may consume up to 50 mA more than ADS127L01, making it less adequate for the intended low-power application. For these reasons, ADS127L01 was selected and implemented in the prototype of the HiFUSPEx.

### 2.5. Digital Signal Processing

Numerous options were considered during the investigation of possible solutions for the digital signal processing (DSP) unit. Each option was analyzed in regard to the following characteristics: power consumption, clock frequency, DSP support, expansion possibilities and ease of implementation. Power consumption generally rises along with the clock frequency of the processor. Similarly, the higher the complexity of the processor (e.g., digital signal processors or field-programmable gate arrays) the longer the development time scales will be.

Based on the comparison of the characteristics from the preceding paragraph and on the comparison of the ADCs using the ARM Cortex-M4 microcontroller unit (MCU), it was decided to use the ARM Cortex solution for the development of the prototype. ARM Cortex devices (currently version M7) offer a very good power consumption vs. clock frequency trade-off. The Cortex Microcontroller Software Interface Standard (CMSIS)—DSP library is being constantly improved, allowing the MCUs to compete with digital signal processors in a growing range of applications. Therefore, the NXP Semiconductor MIMXRT1062DVL6A 600 MHz ARM Cortex-M7 MCU implemented on a Teensy 4.0 development board was chosen for the HiFUSPEx prototype.

## 3. HiFUSPEx—The Prototype

At the core of the prototype lies the DSP unit, which is responsible for a variety of tasks, including digital data processing, user communication, status monitoring and device configuration. The DSP unit chosen for this application can operate with a clock frequency of up to 600 MHz. Combined with the CMSIS—DSP libraries, this enables the execution of formerly computationally expensive functions, such as fast Fourier transform (FFT), in the order of tens of nanoseconds. Moreover, the microcontroller features 1 MB of on-chip RAM that can be utilized for buffering the digitized, sampled audio data. For data transmission, diagnostic and monitoring purposes, there is also a large set of on-chip peripherals.

For instance, the serial peripheral interface (SPI) is employed to transfer the sampled data from the ADC into a buffer located in the RAM. There, the incoming data is organized into 1024 sample blocks effectively covering 4 ms of the input signal. Each 1024-sample long block is then transformed into the frequency domain, where acoustical and electrical compensation functions are applied. Following that, each block goes through a band-pass filter and calibration values are applied. The filtered data is then transferred back to the time domain, where frequency and time weighting, implemented using an IIR filter, are applied to each data block. The one-third octave band filters were implemented using the same approach. It is also worth noting that each completed ADC conversion is followed by the transmission of a 32-bit long SPI data packet. The SPI data packet contains a 24-bit long data sample and an 8-bit long status code, e.g., overflow flag.

By default, the data processed in this way are stored on a 64 GB microSD card as comma separated values. It is also possible to activate raw-data recording using a supplemental computer application. This option is, however, initially disabled for personal data protection reasons. Instantaneous SPL information is provided via a 1.8" LCD screen, where a set of six user-defined SPL values can be viewed. The same set of values, together with the third-octave band SPL values, are also broadcast using a wireless ZigBee protocol. The prototype is equipped with an XBee-S2C communication module for remote live monitoring of the devices. The ZigBee protocol supports addressed, point-to-point, many-to-one communication, enabling multiple ZigBee nodes to report to a single coordinator [28]. In order to monitor the conditions within the prototype’s enclosure, a BOSCH BME680 environment sensor was placed on the main printed circuit board (PCB) [29]. The BOSCH BME680 records air temperature, pressure and humidity information.

It should be noted that SPL measurements can depend highly on external environmental conditions. Although the quality of acoustical components has improved greatly in recent years, the influence of environmental conditions still needs to be considered when estimating the uncertainty budget. They are particularly important when measurements are performed under boundary weather conditions, for instance outdoors on a cold winter day or inside on a hot summer day. For this reason, an environment sensor should be placed at the outer side of the enclosure to provide external monitoring, which is not the case in this version of the prototype.

**Figure 3:**
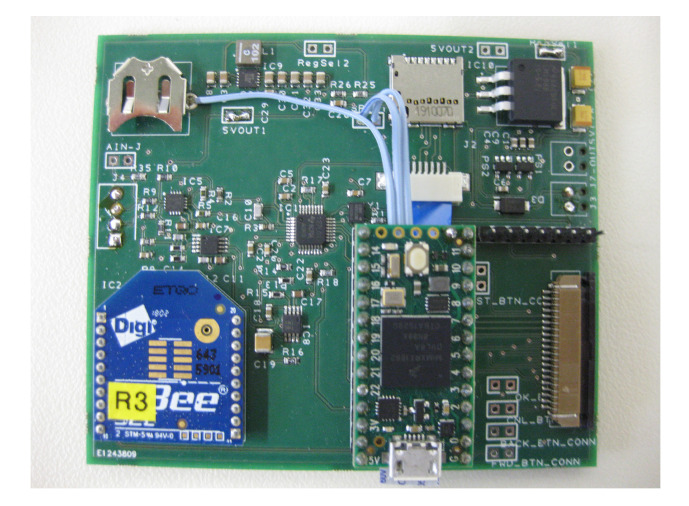
The main printed circuit board including the core elements of the HiFUSPEx prototype. Overall dimensions of the board: 85.2 mm × 72.5 mm.

One of the problems often affecting personal sound exposure meters is the unintentional —or in some cases intentional—corruption of the results. This can easily occur in an industrial setting, where the exposure meter or the microphone may be dropped. In an industrial environment it is also likely that a piece of personal protective equipment (PPE), such as a helmet or a vest, may hit or catch on the microphone. This leads to high SPL values which cannot be traced back to the acoustical events. Therefore, a three-axis STMicroelectronics LIS3DH micro-electro-mechanical system (MEMS) accelerometer is also integrated on the main printed circuit board (PCB). This permits any occurrence of unexpectedly high SPL values to be compared with the accelerometer data in order to identify any sudden position changes. The corresponding time interval can then be excluded from further analysis as it would falsify the overall result of the measurement. In its current position on the PCB, the accelerometer can only account directly for unintended interference with the enclosure of the HiFUSPEx. Therefore, the positioning of the accelerometer should be changed so that it can also detect interference with the microphone. The main printed circuit board comprising the described components is presented in Figure 3.

The prototype is powered by a rechargeable and easily replaceable two-cell lithium-ion battery (7.4 V, 3450 mA). As the average power consumption of the prototype fluctuates around 1 W (the primary factor here is the microphone conditioning module), the capacity of the battery is sufficient for all-day measurements (eight hours). The capacity of the microSD card was also chosen such as to accommodate all-day recording of both raw and filtered data.

## 4. Materials and Methods

The aim of this study was to compare the measurements performed with the HiFUSPEx prototype and those with the USPM. USPM’s performance with regard to the dynamic range and frequency response was known and sufficiently analyzed. Moreover, the device partially fulfills the requirements of the IEC 61672-1:2013 [2] international standard for sound level meters and its response to acoustical signals of frequencies up to 100 kHz is well documented [23]. Therefore, in the light of lacking normative requirements for airborne ultrasound measurements the USPM was deemed as a suitable reference for the intended measurements.

For this purpose, a measurement setup was built within a scanning unit, with an integrated free-field environment, at the PTB in Braunschweig, Germany. In this case, real sound fields are not suitable because the stabilization of sound fields is problematic (e.g., change of interference patterns due to frequency drift caused by the warm-up phase of the machine) [22]. A stable and reproducible test environment is therefore required. For this purpose, idealized sound fields typical of a welding machine, an ultrasonic cleaning bath, and a compressed air gun were created. They show the most important features without being overly complex, and they are sufficiently stationary in time and location.

As previously mentioned in Section 1.1, prolonged exposure to ultrsonic noise has been linked to hearing related health problems. Therefore, airborne ultrasound measurements should be performed in close proximity to worker’s ears. According to the specifications of ISO 9612:2009 [1] for the measurement of occupational noise exposure, the microphone should be placed at the distance of at least 10 cm from the entrance of the external ear canal, and approximately 4 cm above the shoulder. On the other hand, the requirements of the VDI 3766:2012 [4] guideline for the determination of ultrasound exposure at a workplace state that ultrasound noise exposure should be measured directly at the ear, and below the minimum distance of 10 cm. However, this is not always practicable due to obstructions posed by the worker’s PPE. Therefore, all measurements in this work were performed at exactly 10 cm from the worker’s ear. Possible obstructions posed by the worker’s hair and PPE were taken into consideration. To this end, the sound fields around the worker’s ears were initially characterized using the USPM integrated into the scanning unit. This was followed by a set of scans performed around each ear with the HiFUSPEx prototype, the aim being to obtain a direct comparison with the USPM. The scanning unit provides a stable test platform where reproducible measurements may be performed under controlled environment conditions to compare measurement performance of the HiFUSPEx prototype with that of high precision laboratory equipment.

### 4.1. Scanning Unit

The scanning unit was constructed as an open-top cuboid with aluminum frames measuring 2.30 m × 2.45 m × 2.40 m (width × depth × height). Coordinate axes defined parallel to the cuboid frames so that the positions of the scanner can be tracked were assigned as follows: x-axis—width, y-depth and z-axis—height. The scanning operation of the unit was enabled through a motor-controlled 2.20 m long rod, attached to the carriage of the scanner along the z-axis. The movement along each axis was controlled by a two-phase Isel MS 200 HT-2 stepper motor either directly—z-axis—or through a toothed belt (x- and y-axis) [30]. Given the motor’s step angle of 1.8 and the pitch of the ball screws, the effective linear step size was equal to 25 μm.

A free-field environment was created within the scanning unit by lining each side and the bottom of the cuboid with PROTECT R sound insulation panels. These 50 mm thick panels were made from open-cell foam based on BASF Basotect melamine resin. The top of the cuboid was left open. For the purposes of the investigations described in this work, the bottom side was covered with medium-density fiberboard (MDF) plates in order to simulate a reflective surface normally found on factory shopfloors. A more general and detailed description of the scanning unit was previously presented by Schöneweiß et al. [22].

### 4.2. Measurement Setup

As noted above, three different idealized sound fields (typical welding machine, ultrasonic cleaning bath and compressed air gun) were simulated within the scanning unit of the PTB.

#### 4.2.1. Idealized Sound Field of an Ultrasonic Welding Machine

**Figure 4:**
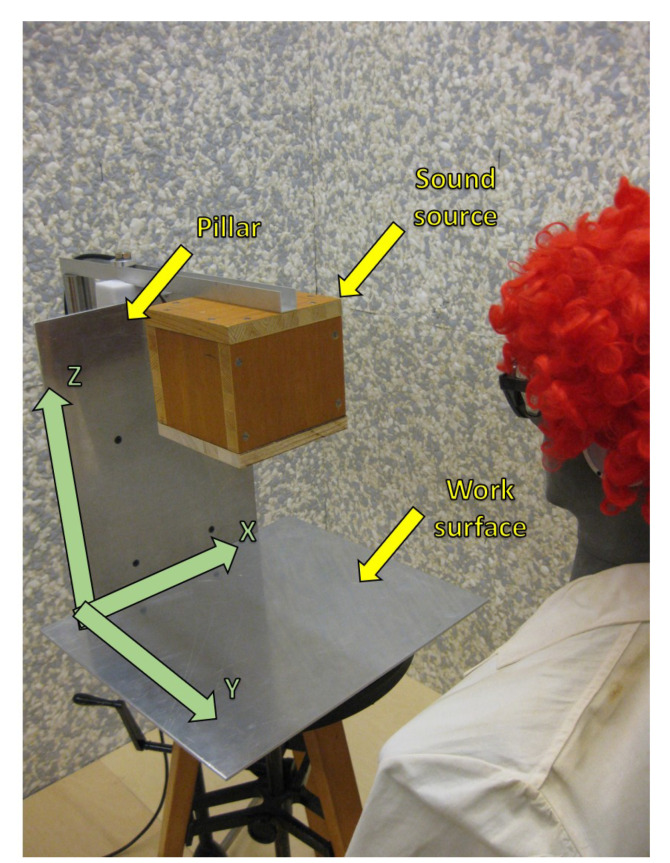
Experimental setup used for the simulation of an idealized sound field produced by an ultrasonic welding machine. The setup was built within the free-field environment at PTB in Braunschweig, Germany.

Due to the complexity and unstability of ultrasound fields produced by industrial welding equipment, an ultrasound field of a welding machine was simulated in the experimental setup shown in Figure 4, with the artificial head facing the sound source and its height adjusted to sedentary position [22,31]. There are individual parts of the simulated welding machine highlighted in the figure together with arrows in green marking directions of XYZ-axes within the scanning unit. Please note that they do not indicate the origin of the scans performed. This is an idealized scenario where artificially generated signals, based on the specification found in the German national guideline VDI 3766:2012 [4], were fed through the loudspeaker. The characteristics of the signal, such as directivity, its contents, dynamic range and frequency were specified in such a way that the signal resembles the one of a real welding machine as closely as possible, and at the same time is easily reproducible.

The arrangement of the simulated welding machine was adapted to the HiQ SOLID PropControl series of welding machines by Hermann Ultraschalltechnik GmbH & Co. KG. The placement of the individual elements of the simulated setup was chosen such as to resemble the actual welding machine as closely as possible, and a comparable sound field was produced. A real welding machine was not used in this study, due to stability problems previously discussed in Section 4. The working frequency of 20 kHz was selected because this frequency potentially causes most of the problems in work environments [17]. The aim was to cover the high-frequency audible and the low-frequency ultrasonic range because most sound energy at the workplace is emitted in this frequency range [17,32]. Moreover, the HiQ SOLID PropControl welding machine with its working frequency of 20 kHz had been the subject of a previous investigation, the results of which provided a good indicator for the expected sound field [22].

The sound source selected for the experiment was a ScanSpeak D2104/712000 tweeter fully enclosed in a wooden box measuring 170 mm × 174 mm × 174 mm. The wooden box was attached to the front end of the arm with the membrane of the tweeter facing the aluminum work surface. The rear end of the arm was screwed to the top of a microphone stand together with a counterweight to provide stable positioning of the tweeter box. The linear distance between the center of the tweeter’s membrane and the aluminum work surface was 30 cm. The work surface was imitated by a 50 cm × 45 cm (width × depth) aluminum plate. Another aluminum plate was placed perpendicularly to and at the rear end of the simulated work surface in order to emulate a pillar of a welding machine. The pillar plate extended from the work surface to the area behind the tweeter box. In this way, any sound wave produced by the tweeter should follow a path similar to the one taken by a sound wave emitted from a real welding machine, i.e., the sound waves should be reflected by two surfaces.

In order to simulate a real work situation, an artificial head was placed centrally in front of the sound source, initially imitating a worker in a sedentary position. The linear distance between the artificial head’s shoulder and the sound source was set to 60 cm. The artificial head faced the center of the sound source with the ear canal positioned 10 cm above the membrane of the tweeter. In line with the specifications included in the ISO 9612:2009 [1], both the sedentary and standing positions of the worker were investigated. The sedentary position was defined with the ear canal at the vertical distance of 1.21 m from the floor level, and the standing position with the ear canal at the vertical distance of 1.55 m from the floor level. The artificial head was placed on a microphone stand and dressed in line with standard safety precautions in an industrial setting, i.e., wearing a coat and safety goggles. In addition, a wig was placed on the artificial head in order to account for possible obstructions when seeking the optimal measurement location.

#### 4.2.2. Idealized Sound Field of an Ultrasonic Cleaning Bath and Compressed Air Gun

**Figure 5:**
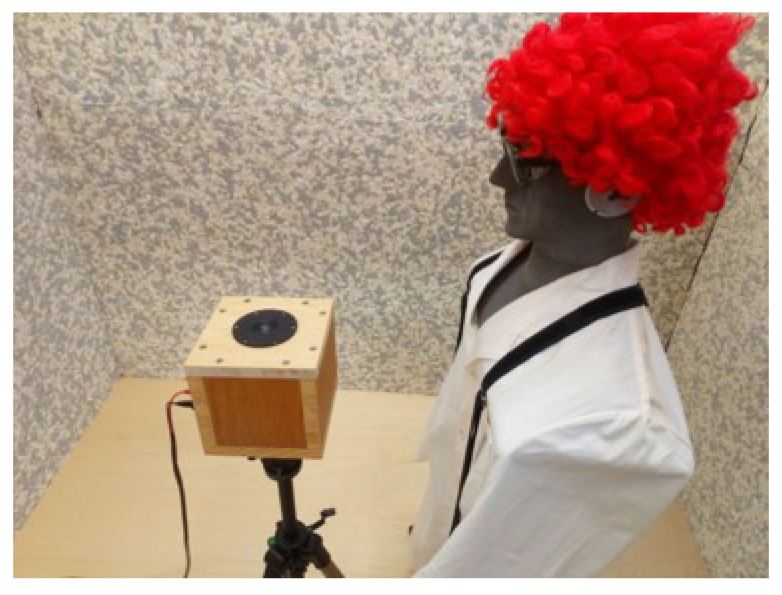
Experimental setup used for the simulation of an idealized sound field produced by an ultrasonic cleaning bath and a compressed air gun built within the free-field environment at the PTB Braunschweig, Germany.

In a similar way, an experimental set-up was organized for the investigation of an idealized sound field of an ultrasonic cleaning bath and a compressed air gun. In this case, however, the sound source was attached to the top of a customized camera stand and positioned to face upwards. For both arrangements—ultrasonic cleaning bath and compressed air gun—the sound source was placed 20 cm below the ear canal of the artificial head at sedentary position height, as presented in Figure 5. In each case, an appropriate signal was generated based on the examples provided by the German national guideline, VDI 3766:2012 [4]. The real signal produced through the generation chain was limited by the characteristics of the ultrasonic loudspeaker, and as such differed from the examples shown in the guideline.

### 4.3. Ultrasound Source

**Figure 6:**
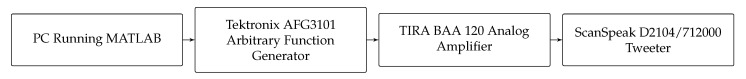
Block diagram of the signal generation chain.

The sound source chosen for this work was the ScanSpeak D2104/712000 tweeter, which provided a very stable SPL value up to the frequency of 40 kHz [33]. The tweeter was fed a signal generated through a signal generation chain as shown in Figure 6. Each signal fed through the chain was defined and verified in a MATLAB script. It was then saved as a *.wfm file, as required by the Tektronix AFG3101 Arbitrary Function Generator, with the defined number of 10,000 points where one complete period of the signal was specified. A signal generated in this way was then fed through a TIRA BAA 120 Analog Amplifier capable of providing a maximum output power of 120 VA for typical audio signals. In this case, however, the output power was maintained at low levels as long as the measured SPL was above the typical values occurring at industrial sites in Germany, thus providing for stable operation across the frequency range of interest [17]. It was in this way ensured prior to the scanning measurements that the SPL of varied-frequency signals—to be used to simulate the idealized sound fields of ultrasonic industrial equipment—was at least 20 dB above the noise floor level of the measurement device. Each signal was also probed for stability by means of 30-min long measurements performed prior to the commencement of the scanning routine. Moreover, during the scanning routines themselves, the SPL at the center of the artificial head’s ear canal was measured multiple times at different stages of the scanning process. This served as a reference to check the stability of the sound source during the scanning process.

#### 4.3.1. Simulated Signal of an Idealized Welding Machine

**Figure 7:**
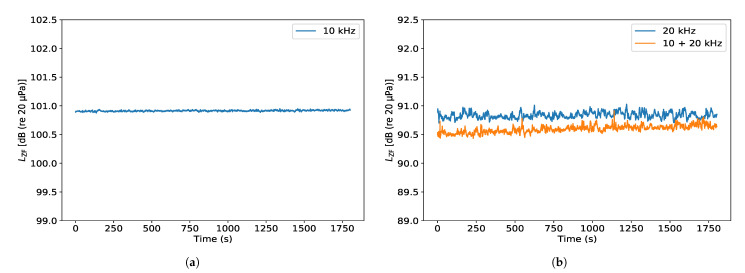
Sound pressure level recorded over the period of 30 min, at the distance of 10 cm away from the loudspeaker, for the following signals (**a**) 10 kHz sine wave and (**b**) 20 kHz sine wave and 10 kHz + 20 kHz multi-sine wave.

The aforementioned welding machine operates at 20 kHz. However, the SPL at the operational frequency is typically accompanied by high SPL harmonic and sub-harmonic contents. When ultrasonic noise exposure in a workplace is considered, the sub-harmonics present in the hearing range are of particular interest. These have been often associated with a negative influence on workers’ hearing. When performing ultrasound measurements, it is therefore vitally important to always monitor the audible sound spectrum as well. For this reason, three different signals were fed through the generation chain in order to test the prototype’s capability to measure both audible and ultrasonic noise. The first signal is a 10 kHz sine wave, simulating the first sub-harmonic of a fundamental working frequency of 20 kHz. The second measurement series contains a 20 kHz sine wave, the fundamental frequency. The third series consists of a multi-sine wave—the sum of the 10 kHz and 20 kHz sine waves. This multi-sine wave was used to represent a typical signal emitted from the welding machine, with the Z-weighted SPL of the 10 kHz signal being 10 dB lower than the corresponding SPL of the 20 kHz sine wave. Figure 7 presents the Z-weighted SPL values recorded over a period of 30 min for the three signals of interest. The highest difference recorded between the maximum and minimum Z-weighted SPL values for the target signals was 0.4 dB over the 30-min measurement interval.

The small fluctuations visible in Figure 7 can be traced back to the stability of the signal generation chain. The first two parts of the block diagram in Figure 6 digitally generate a low power signal of desired characteristics. The electrical signal is then amplified and transduced into an acoustical signal by the loudspeaker. The active components in the amplifier attempt to maintain the specified amplitude and frequency of the signal. However, as the signal fluctuates slightly due to the losses in passive parts of the generation chain small changes are expected. Amplitude changes of up to 0.4 dB over the measured period of 30 min show a sufficient stability for the intended measurements. However, as further measurements could take up to 4 h, additional stability “point check” was used, where the scan area was divided into smaller sections, and the SPL at the origin of the scan was probed after scanning of each of the smaller sections was completed.

#### 4.3.2. Simulated Signal of an Idealized Ultrasonic Cleaning Bath and Compressed Air Gun

Signals emulating the idealized sound fields of an ultrasonic cleaning bath and a compressed air gun were generated according to German national guideline VDI 3766:2012 [4]. The sound field of an ultrasonic cleaning bath was simulated with a working frequency of 26 kHz. This gave rise to a sub-harmonic tonal signal of 13 kHz and a corresponding harmonic of 52 kHz. The high-frequency tonal components were accompanied by a relatively high level of noise across the complete frequency range of the loudspeaker. Due to the limitations of the loudspeaker used, the typical broadband noise signal was not observed above the 31.5 kHz one-third octave band.

The signal generated for the simulation of the idealized sound field of a typical compressed air gun was very similar to the one produced for the ultrasonic cleaning bath. In the case of the compressed air gun, however, the generated signal contained solely a broadband noise signal of sufficiently high SPL. This provided a very homogeneous sound field typical for a compressed air gun. As before, the limitations of the loudspeaker used must be considered in the high-frequency part of the spectrum.

### 4.4. Measuring Equipment

Two measuring devices capable of operating within the ultrasonic frequency spectrum were employed in this study. Initially, the sound field around the worker’s ears was surveyed with the USPM. Then a set of scanning measurements following the exact same scanning patterns was performed with the HiFUSPEx prototype.

#### 4.4.1. USPM Integrated within the Scanning Unit

All measurements utilizing the USPM integrated within the scanning unit were conducted in line with the measurement procedure established by Schöneweiß et al. [22] and with the microphone pointing towards the sound source. The ultrasound measurement procedure also specifies that the measurements be performed with the protection grid removed in order to avoid the grid’s impact on microphone frequency response. However, measurements with the protection grid removed are often not practicable in an industrial setting. Therefore, a series of measurements was also performed with the protection grid installed in order to compare its influence on the measurement results.

**Figure 8:**
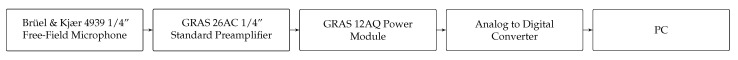
Block diagram of USPM that was used to characterize the sound field around worker’s ears.

A block diagram of the USPM is shown in Figure 8. The GRAS 12AQ Power Module provided the 200 V polarization voltage for the Brüel & Kjær 4939 Free-Field Microphone, and the ±60 V supply voltage for the GRAS 26AC 1/4${}^{\prime \prime }$ Standard Preamplifier [34,35,36]. The gain of the power module was set to 20 dB and the filter characteristics were set to linear response with a high-pass filter of 20 Hz. Detailed description of the USPM can be found in the recent work by Wächtler et al. [23].

The output of the GRAS 12AQ Power Module was connected to an ADC module operating with a sampling frequency of 192 kSPS and 24-bit resolution. This was a standard USPM ADC that was integrated into the control software of the scanning unit. As such, the digitized output signal of the ADC was saved as individual raw data (a *.wav file) for each point within the scanning unit. USPM software was then used in "Offline Analysis" mode to provide time and frequency analysis of the raw data [23].

#### 4.4.2. HiFUSPEx Integrated within the Scanning Unit

In order to perform a direct comparison between the two measurement devices, the HiFUSPEx prototype was integrated into the scanning unit in much the same manner as was the USPM. A detailed description of the device, together with its block diagram, was presented in Section 2. As in the case of the USPM, the protection grid was removed from the microphone’s cartridge for the majority of the measurements. Only in one arrangement was the protection grid left on in order to investigate the influence of the grid on measurement performance. Since both the USPM and the HiFUSPEx prototype use 1/4" microphones, the same holder was used for measurements with the prototype. The length difference between the two microphones was taken into consideration, and the positioning of the arm of the scanning unit was adjusted accordingly.

The data acquisition system of the prototype was customized for this study to allow the time and frequency weighted data to be transferred in real time to a host PC via an RS-232 serial interface. The raw data recorded during the measurements was stored locally on the prototype’s microSD card as *.wav files. For each generated signal, the same scanning patterns used with the USPM were followed.

A detailed description of the device, together with its schematic diagram, was presented in Section 2. As in the case of the USPM, the protection grid was removed from the microphone’s cartridge for the majority of the measurements. Only in one arrangement, the protection grid was left on, in order to investigate the influence of the grid on the measurement performance.

### 4.5. Calibration

**Figure 9:**
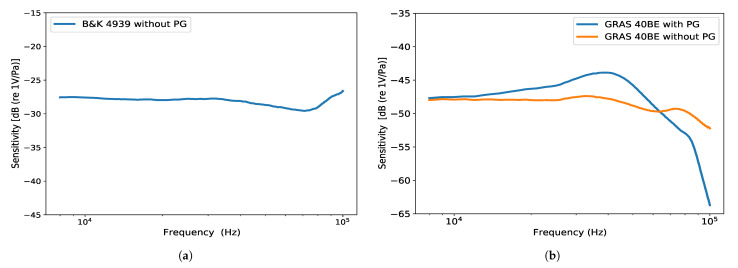
Frequency response of the microphones used in this study (**a**) The frequency response of B&K 4939 microphone, when measured with the protection grid (PG) and (**b**) The frequency response of GRAS 40 BE microphone, when measured with and without PG.

Both microphones used in this study were acoustically calibrated with traceability to the national standard at PTB. A substitution calibration method was employed using two primary calibrated microphones as reference. The sensitivity of the microphones at a 0 incidence angle was determined, with a frequency resolution of 1 kHz, in the frequency range between 8 kHz and 100 kHz. The frequency response of the remaining electrical measuring chain was calibrated at the same frequencies. The final frequency response of the calibration for both microphones is shown in Figure 9. The frequency response of the Brüel & Kjær 4939 microphone is presented only for the configuration having no protection grid mounted. The stability of the measuring chain was repeatedly tested with a Brüel & Kjær Sound Calibrator Type 4231, which was also calibrated to the national standard at the PTB [37].

### 4.6. USPM Measurements

**Figure 10:**
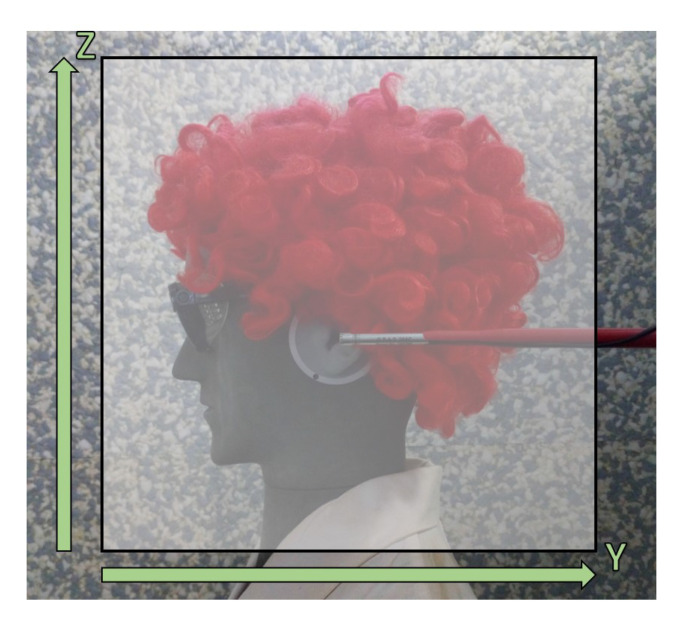
A view from the left side of the artificial head with the scanning area marked by the black frame. Scanning axes together with the USPM microphone at the origin of the scans are also shown.

The measurements performed within the scanning unit at the PTB were primarily used to survey the sound field produced by the ultrasound source in each simulated scenario. For each generated signal, the position of the artificial head (standing or sedentary) as well as the side of the measurement (left or right) were varied. So four scans were performed for every emulated signal for a total of 26 scans (20 scans with no protection grid installed and 6 scans with the grid mounted on the microphone’s cartridge). The scans were performed in yz-plane at a linear distance along the x-axis 17 cm from the center of the artificial head (10 cm away from the entrance of the external ear canal). The distances along the y- and z-axis totaled 32 cm. The origin of the scans (0,0) was placed at the center of the entrance to the ear canal on the yz-plane. As a result, the scans along the y-axis were performed between −16 cm and 16 cm, whereas the scans along the z-axis were done between −14 cm and 18 cm. The vertical distance (along the z-axis) between the artificial head’s shoulder and the microphone tip at the nearest measured point was 3 cm. The vertical distance of the scan was chosen such that it covers the highest point of the worker’s head (including hair). The mapping of the area along the y-axis spanned from the point 5 cm in front of the worker’s shoulder to a point 5 cm behind the worker’s back. The scanning area covered during measurements is presented together with z- and y-axis in Figure 10. Also in Figure 10 the USPM microphone is shown pointing towards the sound source with its tip at the origin of the scanning area. This area was used to complete all the measurements performed in this study.

Each scan was performed with 10 mm resolution in order to provide an outline of the sound field around the worker’s ear. Given that one of the aims of this study was to perform a direct comparison between the two measurement devices, it was attempted to keep the acoustical and environmental conditions within the scanning unit as similar as possible. Therefore, the measurements with both devices were performed on the same day. Due the high frequency of the signal, it was possible to limit the actual measurement time to as little as 2 s, with an additional pause of 3 s to allow the arm of the unit to return to a standstill. With the additional time required for the movement of the arm to the next position, the overall time for one scan was approximately 3 h. Scanning measurements with the USPM were always scheduled for the afternoon, following a scanning session with the HiFUSPEx prototype in the morning.

### 4.7. HiFUSPEx Measurements

The same experimental setup and the same generated signals were used to test the HiFUSPEx prototype. This time the HiFUSPEx was integrated within the scanning unit, which involved replacing the microphone, as presented in Figure 10, and adjusting the control program. As the length of the microphone, placed in the holder within the scanning unit, of the HiFUSPEx was 0.9 cm larger than the one of the USPM, this difference was considered, when preparing the scanning patterns. Exactly the same 26 scans were performed with the HiFUSPEx prototype as described in the preceding section for the USPM. Due to the prototype’s different warm-up procedure, the length of the measurement in each position was set to 5 s with a 3 s pause for the unit to return to a standstill. The overall time of a single scan was therefore accordingly longer for the HiFUSPEx—4 h.

## 5. Results

The results of the comparison of the two measurement systems investigated in idealized simulated scenarios of various industrial settings are presented graphically and numerically below. Due to significant differences in the acoustical components that make up distinct ultrasonic fields, each idealized industrial scenario is considered separately here. The results obtained with both measurement systems for each ultrasonic scenario simulated are first presented in the form of a 2D scan of the measured sound field. These are then used to present the difference between the measurements of the USPM and the HiFUSPEx prototype. Additionally, an average deviation between the two devices for each scanning set-up was calculated. Three different industrial scenarios were simulated: a welding machine simulation (measured without and with the protection grid mounted on the microphone); a compressed air gun simulation (measured without the protection grid); and an ultrasonic cleaning bath simulation (measured without the protection grid).

### 5.1. Ultrasonic Sound Source

Sound fields generated in this work were realized based on previous experience in working with real airborne ultrasonic fields [17,22]. A sound field normally produced by ultrasonic industrial equipment is often extremely unstable, and great effort is required to achieve workable repeatability. For the requirements of this study, it was therefore decided to realize an idealized airborne ultrasound field that would be representative of similar type industrial equipment and stable enough to achieve the required repeatability. Given the results presented in Figure 7, the ultrasonic loudspeaker used presents sufficient stability when generating ultrasonic fields at the required SPL and signal frequency of interest. Moreover, the sound fields generated in this study and discussed in detail in the following sections exhibit characteristic similarities (such as interference patterns) with the previously investigated sound field of an ultrasonic welding machine [22]. Therefore, the sound fields generated here were deemed representative of the ultrasonic equipment typically found at industrial workplaces and hence suitable for the purposes of this work.

### 5.2. Welding Machine—Without Protection Grid

**Figure 11:**
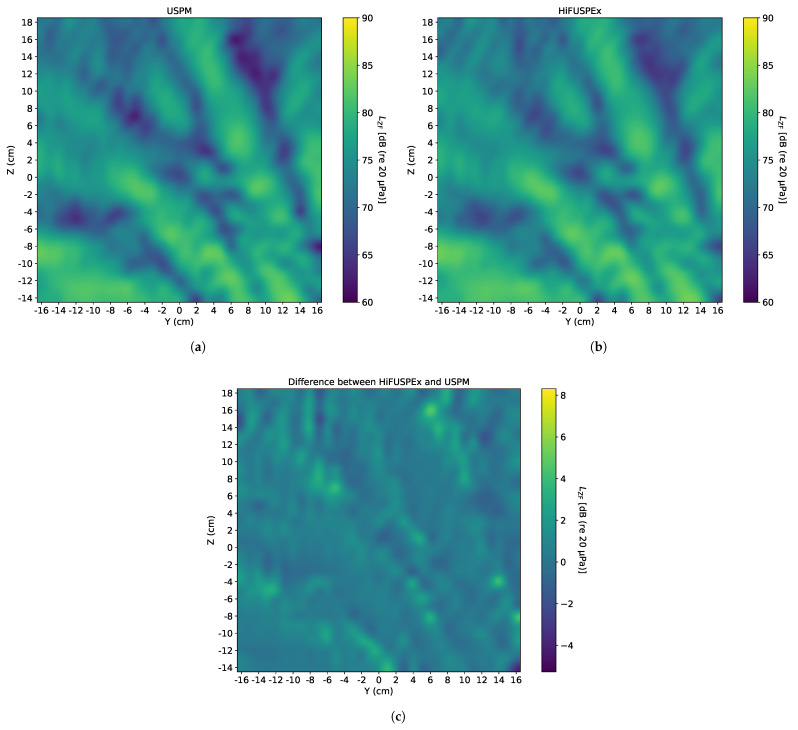
Sound fields within the scanning unit when an idealized signal of a welding machine (10 kHz) was simulated. Results were obtained on the right-hand side with the artificial head in the sedentary position and without the protection grid mounted. The presented plots show the sound fields obtained for: (**a**) USPM; (**b**) HiFUSPEx; and (**c**) difference between the HiFUSPEx and the USPM. Please refer to Figure 10 for identification of ZY-axes.

Since ultrasonic industrial equipment is often accompanied by the high level first sub-harmonic of the working frequency (20 kHz), a 10 kHz sinewave of 90 dB (re 20 μPa), when measured directly over the center of sound source (at 0) at a vertical distance of 1 m, was generated first. The SPL of the 10 kHz sinewave was chosen to be approximately 10 dB (re 20 μPa) below the working frequency of the ultrasonic device, as shown on an exemplified frequency spectrum of a welding machine in VDI 3766:2012-09 [4]. The sound fields imaged by the USPM and the HiFUSPEx prototype are presented in Figure 11a,b, respectively. Additionally, a differential sound field—where the SPL of each point measured with the USPM was subtracted from the value given by the HiFUSPEx (HiFUSPEx—USPM)—is shown in Figure 11c.

**Figure 12:**
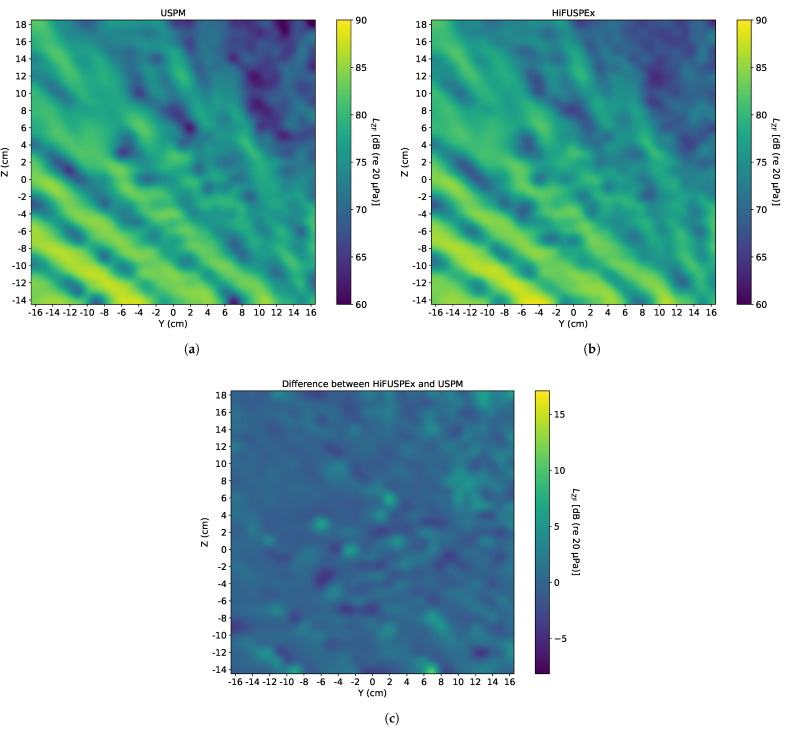
Sound fields within the scanning unit when an idealized signal of a welding machine (20 kHz) was simulated. Results were obtained with the artificial head in the sedentary position and without the protection grid mounted. The presented plots show the sound fields obtained for: (**a**) USPM; (**b**) HiFUSPEx; and (**c**) difference between the HiFUSPEx and the USPM. Please refer to Figure 10 for identification of ZY-axes.

In a similar way, a sinewave of 20 kHz and SPL of 100 dB (re 20 μPa)—when measured directly over the center of the sound source at a vertical distance of 1 m—was generated to investigate the sound field produced when a working frequency of the welding machine was simulated. Analogically to the figure above, the sound fields imaged by the USPM and the HiFUSPEx and the difference between the two devices (HiFUSPEx—USPM) are presented in Figure 12.

**Figure 13:**
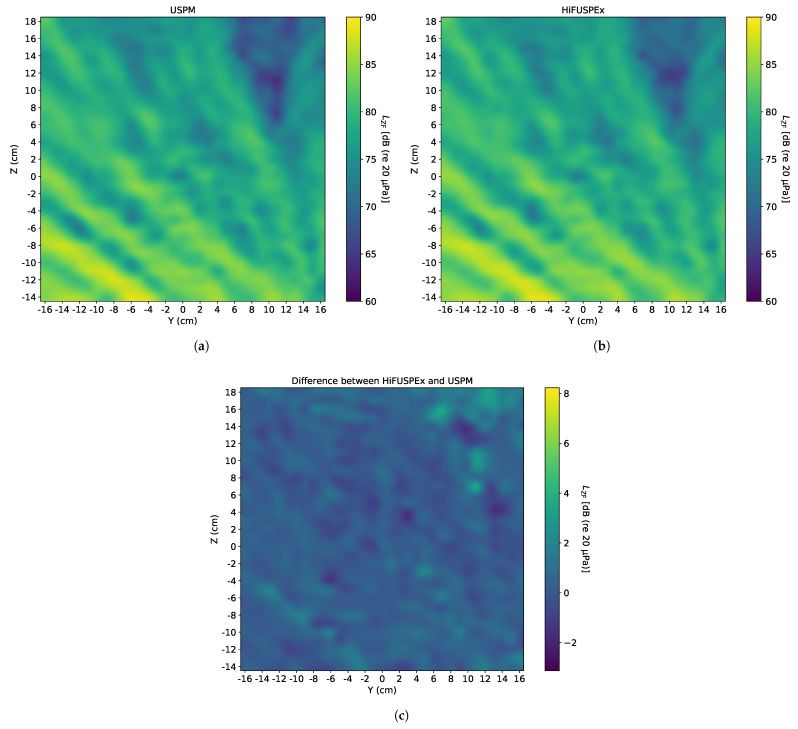
Sound fields within the scanning unit when an idealized signal of a welding machine (10 kHz + 20 kHz) was simulated. Results were obtained on the right-hand side with the artificial head in the sedentary position and without the protection grid mounted. The presented plots show the sound fields obtained for: (**a**) USPM; (**b**) HiFUSPEx; and (**c**) difference between the HiFUSPEx and the USPM. Please refer to Figure 10 for identification of ZY-axes.

Finally, the two signals presented above were combined into a multi-sine signal with a working frequency of 20 kHz and the first sub-harmonic of 10 kHz. As indicated above, the SPL of the working frequency signal was kept at the level of 10 dB (re 20 μPa) above the first sub-harmonic. The results obtained in this case are presented in Figure 13, in the same order as in the two preceding figures, with the differential sound field (HiFUSPEx—USPM) presented in Figure 13c. Additionally, mean difference between results obtained with the two measurement devices together with the extended measurement uncertainty (k = 2, confidence level of 95%) according to the Guide to the Expression of Uncertainty in Measurement (GUM), was calculated. The combined results for all three signals generated within the idealized set-up of a welding machine are shown in Table 1. These results only refer to measurements performed with no protection grid mounted on the microphone.

### 5.3. Welding Machine—With Protection Grid

**Figure 14:**
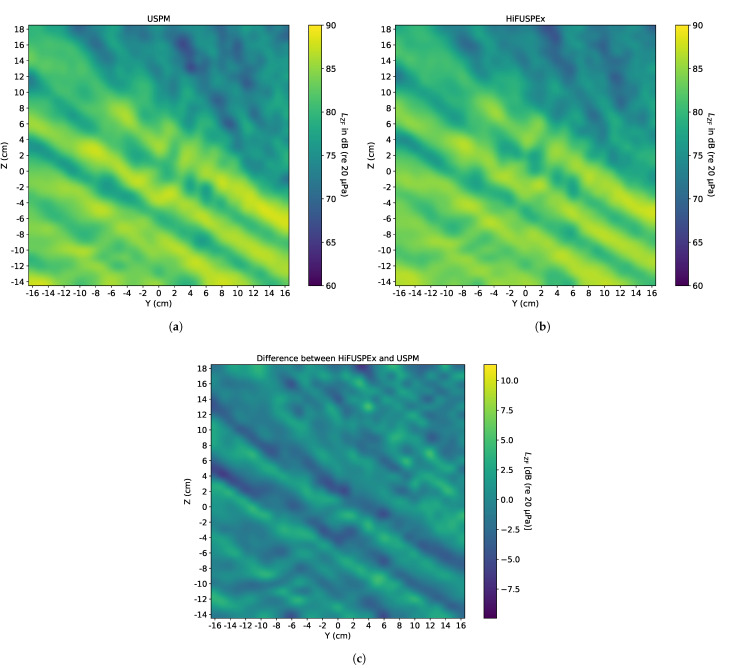
Sound fields within the scanning unit when an idealized signal of a welding machine (10 kHz + 20 kHz) was simulated. Results were obtained on the right-hand side with the artificial head in the standing position and with the protection grid mounted. The presented plots show the sound fields obtained for: (**a**) USPM; (**b**) HiFUSPEx; and (**c**) difference between the HiFUSPEx and the USPM. Please refer to Figure 10 for identification of ZY-axes.

The measurement regime presented in the preceding subsection was then repeated with the protection grid mounted on the microphone. Moreover, only those sound fields mapped around the simulated worker’s head when in the standing position are considered and presented in this subsection, the aim being to compare the characteristics of the sound fields produced after repositioning the artificial head to those of the sound fields observed in the preceding subsection. The order of the individual images is kept as before: the USPM in Figure 14a, the HiFUSPEx prototype in Figure 14b, and the difference between the two devices (HiFUSPEx—USPM) in Figure 14c. Analogically to the measurements with no protection grid, the mean difference between results obtained with the two measurement devices and the corresponding extended measurement uncertainty were calculated for each simulated signal. Measurements with the protection grid on the microphone cartridge were only performed with the artificial head at standing position height. The results obtained for the case considered here are shown in Table 2.

### 5.4. Compressed Air Gun—Without Protection

**Figure 15:**
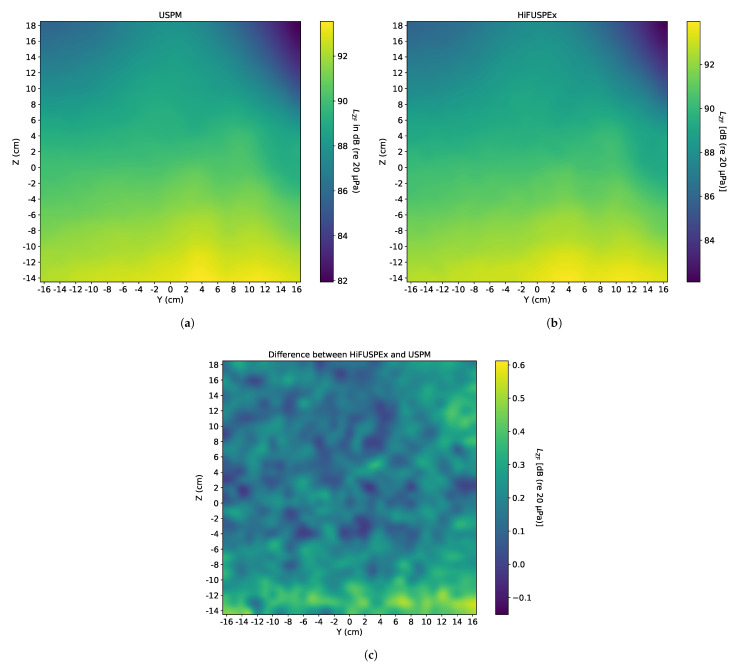
Sound fields within the scanning unit when an idealized signal of a compressed air gun was simulated. Results were obtained on the right-hand side with the artificial head in the sedentary position and without the protection grid mounted. The presented plots show the sound fields obtained for: (**a**) USPM; (**b**) HiFUSPEx; and (**c**) difference between the HiFUSPEx and the USPM. Please refer to Figure 10 for identification of ZY-axes.

Another piece of equipment that is often utilized at numerous industrial sites is a compressed air gun. It is often applied for pressurized cleaning of workpieces. The sound produced by the compressed air gun is a broadband noise signal that reaches up to ultrasonic frequency range, as exemplified in VDI 3766:2012-09 [4]. As a result, the sound field produced by such equipment is more spatially homogeneous than in the case of the simulated welding machine, where "discrete" frequencies were present. Sound fields mapped using the two measurement devices, the USPM and the HiFUSPEx prototype, are presented in Figure 15a,b, respectively. The differential sound field of the two systems (HiFUSPEx—USPM) is shown in Figure 15c. The mean difference between the results obtained using both measurement systems, together with the accompanying extended measurement uncertainty, are numerically presented in Table 3.

### 5.5. Ultrasonic Cleaning Bath—Without Protection Grid

**Figure 16:**
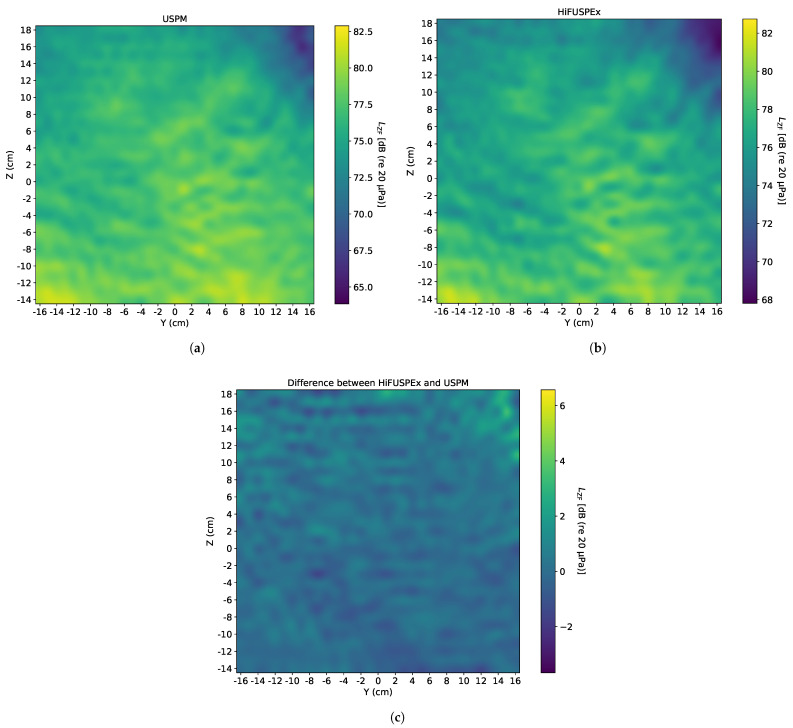
Sound fields within the scanning unit when an idealized signal of the ultrasonic cleaning bath was simulated. Results were obtained in the sedentary position, on the right-hand side, of the artificial head, without the protection grid. Presented plots show sound fields obtained for: (**a**) USPM, (**b**) HiFUSPEx, and (**c**) difference between the HiFUSPEx and the USPM. Please refer to Figure 10 for identification of ZY-axes.

Ultrasonic cleaning baths are also frequently employed at industrial workplaces for high-quality cleaning. Signals associated with the operation of ultrasonic cleaning baths combine a broadband noise signal with high tonal frequencies. In this work a 26 kHz working frequency signal (together with its sub-harmonics, and harmonics) was simulated, as exemplified in the VDI 3766:2012 [4] guideline. The resulting sound fields produced by the USPM and the HiFUSPEx prototype are presented in Figure 16a,b, respectively. The difference between the two measurement systems (HiFUSPEx—USPM) is graphically presented in Figure 16c. A numerical analysis of the measurements performed is also presented in Table 4, where the mean difference and extended measurement uncertainty for results obtained in this simulated scenario are calculated. All measurements were performed without the protection grid mounted on the microphone cartridge.

### 5.6. Distribution Comparison across Various Cases Investigated

**Figure 17:**
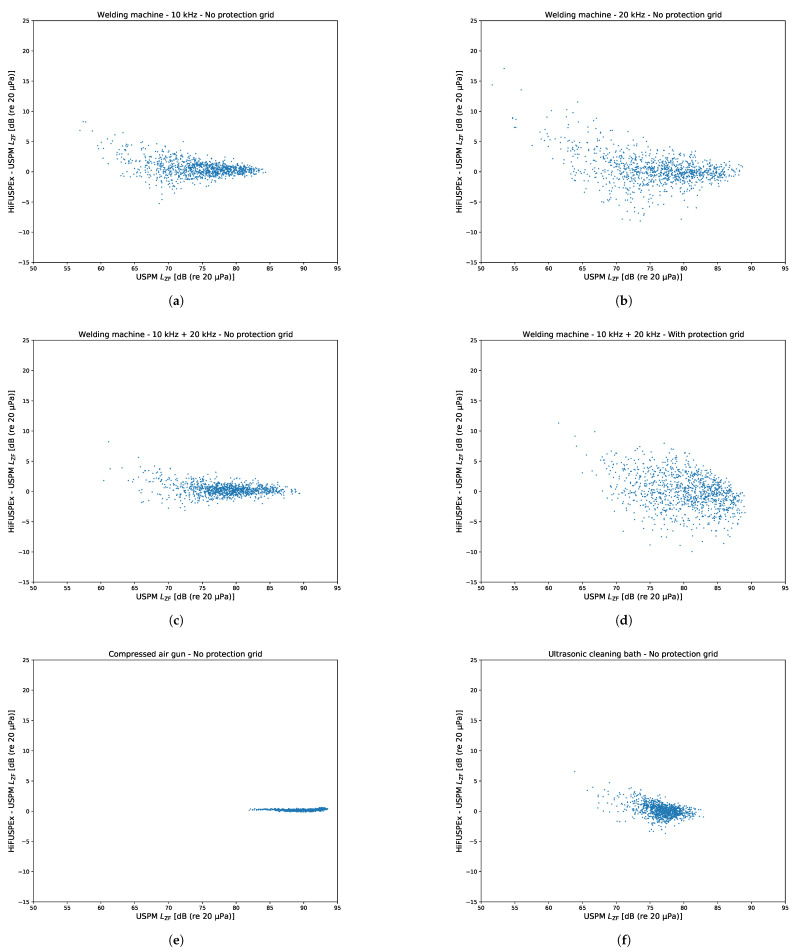
HiFUSPEx deviation from USPM for the selected cases: (**a**) welding machine −10 kHz (no protection grid); (**b**) welding machine −20 kHz (no protection grid); (**c**) welding machine −10 kHz + 20 kHz (no protection grid); (**d**) welding machine −10 kHz + 20 kHz (with protection grid); (**e**) compressed air gun (no protection grid); and (**f**) ultrasonic cleaning bath (no protection grid).

In order to better present the deviation of the measurements performed with the HiFUSPEx prototype from the ones obtained with the USPM another set of distribution graphs was plotted as seen in Figure 17. Here, the SPL measured with the USPM is presented on the *x*-axis. This is plotted against the difference (HiFUSPEx—USPM) on the *y*-axis. Deviation distribution for the cases presented in the preceding subsections are shown in Figure 17a–f.

## 6. Discussion

Depending on numerous factors relating to either the sound source, the generated sound field, or the measurement devices used, there are a number of observations that can be made following closer investigation. To keep this discussion consistent with the preceding section, the individually simulated scenarios will be described here in the same order.

### 6.1. Idealized Sound Field Produced by a Simulated Welding Machine

Based on the results presented in the preceding section, a general trend can be observed. There is a good agreement between the results obtained with both devices in the louder areas but correspondingly higher discrepancies in the quieter parts of the sound fields. This phenomenon can be observed, for instance, in Figure 11c, which presents the sound field drawn from the difference between the results obtained from the HiFUSPEx and the USPM. It can be seen that the image in Figure 11c “mirrors” the sound fields mapped by the measurement devices, and that the interference patterns in both Figure 11a,b are inverted in Figure 11c. The mean difference for the case presented in Figure 11 is 0.56 dB. The extended measurement uncertainty was calculated to be 2.62 dB, which also agrees with the image, where the majority of the 2D scan shows a difference of approximately 2.0–2.5 dB.

When the individual images obtained by the USPM and HiFUSPEx are directly contrasted, as for instance Figure 11a,b, nearly identical patterns can be recognized. However, the SPL levels measured by the HiFUSPEx in the quieter parts of the sound field are significantly higher than those measured with the USPM. And this in turn leads to higher extended measurement uncertainty presented for this case in Table 2.

Furthermore, the influence of the protection grid appears to only be significant when the extended uncertainty values are compared for the corresponding measurements in the welding machine scenario and when the multi-sine (10 kHz + 20 kHz) signal is simulated. In this case, the differences between the corresponding values are 2.66 dB (Standing/Right) and 3.22 dB (Standing/Left). This appears to be in line with the difference seen in the microphone’s frequency response with and without the protection gird, as presented in Figure 9b. There, a steady rise in sensitivity can be observed up to 40 kHz, reaching approximately 3.0–3.5 dB at the highest point. Given that most of the components of the acoustical signals measured in this work lay below this value, this could have an impact on the effective difference between the results of the measurement devices.

It should also be noted that frequency response compensation was adjusted for the two measurements (with and without protection grid) based on the microphone calibration performed prior to the measurements in the scanning unit. As a result, the majority of the differences between measurements with and without the protection grid were compensated by the appropriate frequency response. Moreover, placing the measurement set-up in a practically free-field environment minimized the influence of reflections from other objects normally found in an industrial environment. This means that most of the sound collected by the microphone was reaching it from a very narrow angle directly before it. This would not be the case in an industrial setting, where sound reflections arriving at the microphone from various angles would make the influence of the protection grid more prominent.

When the results obtained in the microphone configuration with and without the protection grid are compared for the other frequencies measured (purely audible 10 kHz and working frequency 20 kHz), the influence of the protection grid is not easily noticeable. Given the high heterogeneity of the sound fields, the most likely reason for the discrepancies can be associated with the specific sound field produced on the day of the measurements. The characteristics of the sound fields produced with both devices on the same day are comparable. However, when the results obtained with the same simulated signal but on different days are contrasted, the specific patterns are altered. This can be easily perceived by examining the dissimilarities seen between Figure 13 and Figure 14 in the upper-right part of the sound fields.

As such, the relatively large extended measurement uncertainty values, e.g., 8.30 dB (welding machine −10 kHz with no protection grid, standing, left ear), are primarily associated with the specificity of sound fields generated. The higher values of the extended measurement uncertainty for any given case suggest that larger parts of the scanned sound fields are covered by the signals of lower SPL. This difference in the quieter areas of the sound fields is caused by the inherent noise floor level present on the prototype’s electronic circuit. This was investigated by recording the SPL using both devices within the scanning unit when there was no acoustical signal generated. The measured noise floor level of the USPM was approx. 34.5 dB (re 20 μPa) ($L_{\mathrm{Aeq}}$) and the noise floor level of HiFUSPEx was approx. 55.8 dB (re 20 μPa) ($L_{\mathrm{Aeq}}$). This is consistent with the deviation graphs shown in Figure 17b, where the maximum deviation is approximately 18 dB.

Another important part of the analysis involves attempting to compare the results obtained from the measurements of the simulated welding machine to the investigation performed by Schöneweiß et al. [22]. Given the principal difference between the two studies—one performed with a real welding machine and the other with an idealized, simulated version of it (the simulated 10 kHz + 20 kHz signal was considered here as it most resembles a real welding machine)—only a partial comparison is possible. Nevertheless, significant similarities between the two studies can be identified.

In both cases, a similar distribution pattern of the sound waves can be observed, e.g., interference pattern. Furthermore, both scenarios reveal a clear pattern of local maxima and local minima which may lie very close to one another. Since the distances between the extremes (SPL difference in the order of tens of dB) can be as little as a few centimeters (e.g., 1–2 cm), the repeatability of the measurements performed with the two devices is crucial. For this reason, extra caution was taken after each measurement with the HiFUSPEx, when the scanning unit was manually adjusted to perform measurements with the USPM. Despite the best efforts at keeping all distances unchanged, the slightest variation may lead to a significant change in SPL. This could in turn further contribute to the outcome of the comparison, especially in the regions at the boundaries between the local extremes. This is one of the key issues that may also have a high impact on the outcome of the performance comparison between the two devices.

### 6.2. Idealized Sound Field Produced by a Simulated Compressed Air Gun

The sound field as well as the corresponding measurement results suggest a much better agreement between the two devices when a homogeneous sound field of sufficiently high SPL is simulated. This can be observed in Figure 15, which presents the results of the sound field mapping of the simulated compressed air gun. The upper part of the differential sound field, Figure 15c, exhibits the same characteristics as the sound field produced by the welding machine. The differences are higher in the quieter parts of the images and lower in the louder parts of the sound field.

The lower part of the differential sound field presented in Figure 15c reveals that there are indeed differences in the louder areas of the sound field. However, it must be taken into consideration that the scale of the difference dropped dramatically. In this case, the mean difference is equal to 0.2 dB with the extended measurement uncertainty of 0.24. The highest difference between the two measurement devices is approx. 0.6 dB compared to approx. 16.0 dB for the idealized sound field of a welding machine.

### 6.3. Idealized Sound Field Produced by a Simulated Ultrasonic Cleaning Bath

When compared with the two previously presented scenarios, the idealized sound field of an ultrasonic cleaning bath combines the characteristics of the welding machine (tonal high-frequency signals) and those of the compressed air gun (broadband noise). As a result, the sound field is more heterogeneous than in the case of the simulated compressed air gun. This is also reflected in the extended measurement uncertainty values in Table 4.

The estimated extended measurement uncertainty values for this case are comparable with the corresponding results obtained in the scenario in which a welding machine with the multi-sine signal (10 kHz + 20 kHz) was simulated. This further supports the previous observation that differences between the two measurement devices are smaller when the investigated sound fields are more homogeneous.

### 6.4. HiFUSPEx and Its Potential for Dependable Ultrasound Measurements

When analyzing the results presented in this work, as well as the outcome of ultrasonic sound field characterization reported by Schöneweiß et al. [22], it becomes clear that ultrasonic exposure at a workplace cannot be measured statically. The way SPL spreads within the ultrasonic sound field is very dynamic, and can be affected by, for instance, industrial equipment in the close proximity to the source of ultrasound emission. Even if every ultrasonic field encountered in industry were characterized through the lengthy scanning process, the movement pattern of a worker cannot be modeled. Individual sound level measurement, reliably accounting for the worker’s mobility, can only be achieved through a portable solution that would move together with the worker. The prototype presented here offers this flexibility coupled with SPL detection performance comparable to that of the USPM.

## 7. Uncertainty Consideration

When evaluating the uncertainty of any measurement system, the GUM should be consulted to identify the relevant methods for uncertainty calculations [38]. In specific cases (for instance, an acoustic measurement such as in this study), international standards and, if applicable, national guidelines must be taken into account if directly relevant uncertainty contributors can be found. Literature sources describing similar assessments attempted in the past are equally important in the evaluation process.

The HiFUSPEx is primarily meant to be deployed for airborne ultrasound monitoring at workplaces, but there are currently no international standards that could be utilized to define the requirements for that frequency range. However, high-frequency audible and ultrasound signals are normally accompanied by sub-harmonic contents that also need to be appropriately identified. Therefore, the IEC 61672-1:2013 [2] international standard was used to test the frequency response of the prototype against the limits specified up to 20 kHz. According to IEC 61672-1:2013 [2], the uncertainty of a sound level measurement needs to be estimated based on the uncertainties associated with the electro-acoustical performance of the sound level meter as well as those directly related to the specific measurement situation. As a result of the former, a chosen frequency weighting, for instance, will have an impact on the overall uncertainty of measurement. The latter suggests that factors external to the sound level meter, such as the stability of the sound source and the measurement procedure, may have a significant influence on the overall uncertainty estimation. The uncertainty of any sound level measurement therefore needs to be considered separately for each specific measurement.

Previous studies that computed an uncertainty budget for ultrasound measurement were also analyzed in order to identify the most relevant uncertainty contributions for the HiFUSPEx. Given the stage of the development of the device, only partial testing of the prototype in its current version was completed. Based on the requirements specified in IEC 61672-1:2013 [2] and in preceding studies where uncertainty estimation was attempted, a procedure based on 24 contributions used by Wächtler et al. [23] was adapted for this study. At this stage, only five electro-acoustical components were included in the calculation of the uncertainty budget [23,38,39,40]. These are marked with asterisks in Table 5. Additionally, the remaining 19 contributors as listed by Wächtler et al. were estimated based on the experience gained from measurements performed with both devices [23]. The expanded electro-acoustical uncertainty estimated in this way with reference to the GUM specification (k = 2, confidence level of 95%) is 1.31 dB.

A comparison with previous studies dealing with the uncertainty of sound level meters according to IEC 61672-1:2013 [2] cannot be easily performed. Not only does the IEC 61672-1:2013 [2] standard not cover frequencies above 20 kHz, it is also intended solely for audible sound level meters. Nonetheless, Wächtler et al. [23] calculated, based on 24 contributing components, the electro-acoustical expanded uncertainty (k = 2, according to GUM) of the USPM to be equal to 1.15 dB for equivalent level quantities. In their work, Wächtler et al. [23] identify electromagnetic interference (EMI) as the most significant contributor to their uncertainty calculation. A study by Radosz [40], on the other hand, used 12 contributing components from IEC 61672-1:2013 [2] for uncertainty consideration and measured ultrasonic signals only up to 40 kHz. This resulted in an expanded uncertainty of less than 1.0 dB. The analysis performed by Radosz [40] excluded electromagnetic interference from uncertainty considerations, which was identified by Wächtler et al. [23] (and was also estimated in this study) to be the highest contributor to the electro-acoustical uncertainty. The study by Bjor [39] on the other hand only deals with audible sound frequencies and yields an expanded electro-acoustical uncertainty of 0.52 dB.

The high contribution of EMI to the electro-acoustical uncertainty of the USPM, as determined by Wächtler et al. [23], can be traced to the "open design" of the system. The USPM consists of a laptop and additional essential equipment linked by cables. Although all these parts are stored in a sturdy carrying case for transportation, the case must be kept open when performing measurements. While USPM’s high contribution from EMI is somewhat expected, it should be possible to reduce this contribution for the HiFUSPEx. Initial testing performed with the HiFUSPEx in its current version, where rudimentary protection was realized using an electromagnetic compatibility (EMC) spray, showed promising immunity against EMI signals of frequencies between 2 GHz and 6 GHz. Given that the enclosure of the prototype will be redesigned to accommodate the required changes in electronic circuitry, a full investigation will be performed upon completion of the final version of the prototype. Despite the expectation of a lower EMI contribution for the HiFUSPEx, the USPM’s high contribution was directly applied in this study as a worst-case scenario estimation.

Although the estimated electro-acoustical uncertainties previously found in the literature in association with ultrasound measurements show values only slightly lower than the one obtained here for the HiFUSPEx prototype, a direct comparison of the different technologies is not possible and extra care must always be taken when interpreting this data. The prototype of the HiFUSPEx is an example of a personal sound exposure meter and as such more suited for assessment against IEC 61252:1993 [3]. However, the requirements specified in this standard only apply up to 8 kHz (with recommendations given up to 12.5 kHz), and the acceptance limits are in line with the requirements of class 2 devices according to the sound level meter standard IEC 61672-1:2013 [2]. Furthermore, class 2 devices cannot be employed to perform sound level measurements that satisfy the requirements of occupational health monitoring. Therefore, the normative demands for class 1 sound measuring devices according to the IEC 61672-1:2013 [2] international standard were used for the evaluation of the prototype’s performance together with the additional requirements specified in the VDI 3766 [4] national guideline. For instance, deviations from the expected values for the frequency weighting test (with A, C and Z weightings) all fall within the limits specified in IEC 61672-1:2013 [2] for class 1 sound level meters.

Further tests are required to fully assess the prototype’s sound level measurement performance—including additional electrical, acoustical and environmental (e.g., temperature, humidity, static pressure) tests—and these tests will undoubtedly have an effect on the uncertainty estimation. Nonetheless, the tests completed to date support the trend observed based on preceding studies that sound level measurements within the ultrasonic frequency range are associated with a higher electro-acoustical uncertainty. The overall expanded uncertainty of a measurement is, however, primarily influenced by the lack of a measurement procedure that accounts for the specific characteristics of airborne ultrasound, and this lack contributes greatly to the uncertainty [22].

## 8. Conclusions and Outlook

This article reports on the requirements for, development of, and initial tests performed with the prototype of a high frequency and ultrasound personal exposure meter (HiFUSPEx). The primary aim of this study was to compare the performance of the HiFUSPEx prototype with that of the ultrasound level measuring system (USPM). The results obtained show that the two devices perform similarly within the corresponding inhomogeneous sound fields. However, deviations between the two systems are more pronounced in the quieter areas of the heterogeneous sound fields due to the inherent noise floor of the HiFUSPEx. When their performance is compared within homogeneous ultrasound environments, the overall agreement between the two devices is significantly enhanced.

The inherent noise floor issue was identified during laboratory testing of the prototype. The main source of the problem was traced to the impedance matching between the output signal of the constant current power supply for the IEPE microphone and the input of the ADC. This is currently being addressed, and the next version of the prototype will include an additional buffering stage between the IEPE power module output and the ADC’s input. To further alleviate the inherent noise problem on the PCB, the arrangement of the electronic components will be optimized, as will all electrical connections and the prototype’s enclosure. Due to the required technical changes, the prototype was only partially tested in its current form. A full evaluation of the optimized version of the prototype in line with the requirements of type approval tests for sound level meters is planned.

Since the idea behind the HiFUSPEx was to provide a solution capable of accounting for the specific properties of airborne ultrasound at workplaces, it includes time and frequency weightings recommended by the VDI 3766:2012 [4] guideline. It also includes ten third-octave band filters which can be manually set to cover either the audible or ultrasonic frequency range. The current limitation to the number of third-octave band filters is dictated by the computational performance of the microcontroller. However, alternative options are being considered for the computational unit which would allow these limitations to be removed.

The portability of the prototype was also only partially examined since testing could only be done under laboratory conditions. The attachment mechanism is currently realized using a belt clip, which allows the device to be slid on to the worker’s belt. Portability testing consisted only of checking the system’s attachment to the artificial head, and then having it worn by laboratory workers during the course of their routine laboratory tasks. The device does not appear to have caused any discomfort to the laboratory workers. An optimized version of the clip will be realized as a multi-purpose fastening solution so that flexible attachment options can be offered as required.

Due to the current placement of the environment monitoring sensor (EMS)—which monitors temperature, humidity and static pressure—and the accelerometer on the main PCB, they can only provide information about the conditions inside the device’s enclosure. It is therefore of paramount importance that the EMS be positioned such that it measures the conditions directly external to the device. The accelerometer, on the other hand, will be arranged together with the microphone holder and placed on the worker’s shoulder.

In the microphone’s current version, it could be placed on the worker’s shoulder, as was done when testing the portability of the device, and its position adjusted to the requirements of sound level monitoring at a workplace as per international standard ISO 9612:2009. But since these requirements were developed for audible sound monitoring, the portable measurement solution described in this paper will be used as a starting point for further investigation into an optimized positioning of the microphone. It follows that a complete measurement and calibration procedure must be prepared for the HiFUSPEx which would take into consideration the specificity of ultrasonic sound fields.

**Figure 18:**
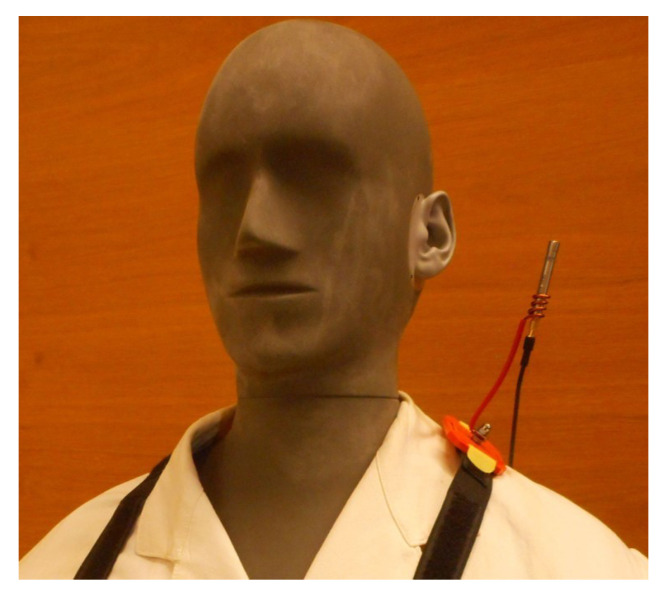
Preliminary placement location for the HiFUSPEx microphone located on the artificial head’s shoulder with a rudimentary microphone holder.

Based on the results presented in this work, as well as in the preceding study by Schöneweiß et al. [22], it is clear that a stationary measurement of airborne ultrasound disregards important characteristics of the sound field. Furthermore, worker’s individual physiology and their mobility are equally ignored. Due to its portability, the HIFUSPEx can account for worker’s physiology and mobility during measurements. As a result, a more realistic estimate of a constantly changing individual ultrasound exposure can be made. However, the optimal placement position of the microphone on the shoulder still remains to be found. It should allow for the best possible instantaneous SPL estimation without limiting worker’s movement or posing a health and safety risk. Moreover, by placing the microphone on worker’s shoulder the prototype is fully capable of reliably performing standard audible SPL measurements according to ISO 9612:2009 [1]. A preliminary location on the worker’s shoulder, where the microphone may be placed is presented in Figure 18. The figure shows a rudimentary microphone holder which was considered for the laboratory trials.

Despite the necessary optimizations described in this section, the device showed a sound level measurement performance similar to that of an ultrasound level meter (within the homogeneous sound field) whose applicability for ultrasound exposure monitoring at workplaces had been previously investigated [23]. It thus bears good potential for providing a solution for airborne ultrasound measurement, which due to the specificity of such sound fields must be performed on a case-by-case basis. With its compact size and ease of use, the device promises to furnish a concept for enabling the much-required deeper investigation into airborne ultrasound at the workplace.

## Figures and Tables

**Table 1:** Mean difference between the HiFUSPEx and the USPM together with the extended measurement uncertainty calculated for each simulated scenario of the idealized set-up of a welding machine. Measurements performed with no protection grid (PG) mounted on the microphone cartridge.

Welding Machine (10 kHz) without PG		
**Ine Position**	**Mean Difference (dB)**	**Ext. Uncertainty (dB)**
Ine Sedentary/Right	0.56	2.62
Sedentary/Left	0.77	3.86
Standing/Right	1.13	3.47
Standing/Left	1.79	8.30
Welding Machine (20 kHz) without PG		
Sedentary/Right	0.33	4.99
Sedentary/Left	0.98	5.72
Standing/Right	1.06	5.45
Standing/Left	0.71	5.95
Welding Machine (10 kHz + 20 kHz) without PG		
Sedentary/Right	0.32	1.91
Sedentary/Left	0.52	2.46
Standing/Right	−0.13	3.04
Standing/Left	0.53	2.57

**Table 2:** Mean difference between the HiFUSPEx and the USPM, together with the extended measurement uncertainty calculated for measurements within the idealized set-up of a welding machine. Measurements performed with protection grid (PG) mounted on the microphone cartridge.

Welding Machine (10 kHz) with PG		
**Ine Position**	**Mean Difference (dB)**	**Ext. Uncertainty (dB)**
ine Standing/Right	0.98	3.90
Standing/Left	1.00	4.83
Welding Machine (20 kHz) with PG		
ine Standing/Right	−0.11	4.26
Standing/Left	−1.08	6.92
Welding Machine (10 kHz + 20 kHz) with PG		
ine Standing/Right	−0.02	5.70
Standing/Left	−0.56	5.79

**Table 3:** Mean difference between the HiFUSPEx and the USPM, together with the extended measurement uncertainty calculated for measurements within the idealized set-up of a compressed air gun. Measurements performed with no protection grid mounted on the microphone cartridge.

Compressed Air Gun		
**Position**	**Mean Difference (dB)**	**Ext. Uncertainty (dB)**
ine Sedentary/Right	0.2	0.24
Sedentary/Left	0.14	0.22
Standing/Right	0.24	0.33
Standing/Left	0.13	0.32

**Table 4:** Sound fields within the scanning unit when an idealized signal of an ultrasonic cleaning bath was simulated. Results were obtained on the right-hand side of the artificial head in the sedentary position and without the protection grid mounted. The presented plots show the sound fields obtained for: (a) USPM; (b) HiFUSPEx; and (c) difference between the HiFUSPEx and the USPM.

Ultrasonic Cleaning Bath		
**Position**	**Mean Difference (dB)**	**Ext. Uncertainty (dB)**
ine Sedentary/Right	0.17	2.08
Sedentary/Left	0.03	1.64
Standing/Right	0.45	3.60
Standing/Left	0.6	2.18

**Table 5:** Contributions to the electro-acoustical uncertainty as computed based on the tests completed with the current version of the prototype and estimated with reference to the uncertainty budget of the USPM [23].

		Equivalent Level Quantity	
**Category**	**Contribution**	**Standard Uncertainty (dB)**	**Index (%)**
Indication	Display restriction	0.029	0.2
Acoustical	Microphone directivity	0.250	14.6
	Protection Grid	0.000	0.0
	Enclosure	0.030	0.2
	Instrument noise	0.070	1.1
Calibration	AD converter freq. response	0.190	8.4
	Acoustical measurement chain	0.140	4.6
	Calibrator sensitivity adjustment	0.100	2.3
Electrical	Frequency weighting	0.300	21.0
	ADC level linearity	0.200	9.3
	Microphone level linearity	0.025	0.1
	Time weighting	0.000	0.0
	Toneburst	0.080	1.5
	Repeated tonebursts	0.070	1.1
	Peak detection	0.000	0.0
	Filter banks	0.100	2.3
	Averaging & Integrating	0.000	0.0
	High-level stability	0.005	0.0
	Continuous-operation stability	0.005	0.0
Environmental	Temperature	0.100	2.3
	Humidity	0.000	0.0
	Static pressure	0.100	2.3
	Electromagnetic interference	0.350	28.6
	Power supply	0.000	0.0
**Electro-acoustical uncertainty**		**0.655**	**100.0**
**Expanded uncertainty**		**1.31**	

## Data Availability

Measurement data collected in this study is publicly available from the PTB Open Access Repository; doi:10.7795/720.20211214.
